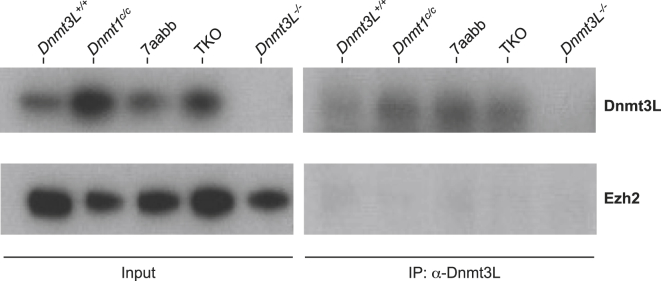# The Dnmt3L ADD Domain Controls Cytosine Methylation Establishment during Spermatogenesis

**DOI:** 10.1016/j.celrep.2015.04.041

**Published:** 2015-05-12

**Authors:** Georgios Vlachogiannis, Chad E. Niederhuth, Salih Tuna, Athanasia Stathopoulou, Keijo Viiri, Dirk G. de Rooij, Richard G. Jenner, Robert J. Schmitz, Steen K.T. Ooi

(Cell Reports *10*, February 17, 2015; 944–956)

In the published version of this manuscript, there was an error in Figure S4. The original scanned image file of the film contained six lanes, with the wild-type sample run in duplicate at either end of the gel. While the sixth lane was cropped out of the image of the film probed with anti-Dnmt3L antibody, it was accidentally included in the image of the film probed with anti-Ezh2 antibody.

The corrected figure is below. The authors regret the error.

**Figure S4:**